# The role of arts-based curricula in professional identity formation: results of a qualitative analysis of learner’s written reflections

**DOI:** 10.1080/10872981.2022.2145105

**Published:** 2022-11-14

**Authors:** James Aluri, Joyce Ker, Bonnie Marr, Heather Kagan, Kaitlin Stouffer, Philip Yenawine, Margot Kelly-Hedrick, Margaret S. Chisolm

**Affiliations:** a Department of Psychiatry and Behavioral Sciences University School of Medicine, University Mental Health, Baltimore, Maryland, USA; b Department of Medicine, Science, Humanities at Johns Hopkins University in Baltimore, Baltimore, Maryland, USA; c Section of Palliative Medicine Division of General Internal Medicine Johns Hopkins University in Baltimore, Baltimore, Maryland, USA; d Department of Medicine at Memorial Sloan Kettering Cancer Center with a Secondary Appointment as an Instructor at Weill Cornell College of Medicine Maryland, USA; e Johns Hopkins University, Baltimore, Maryland, USA; f Visual Thinking Strategies and an Independent Writer and Educator, Baltimore, Maryland, USA; g Duke University School of Medicine, Durham, North Carolina, USA; h Department of Psychiatry and Behavioral Sciences, Department of Medicine, Johns Hopkins University in Baltimore, Baltimore, Maryland, USA

**Keywords:** Professional identity formation, health humanities, reflective writing, visual Arts-Based learning, visual thinking strategies

## Abstract

**Background:**

Professional identity formation is an important aspect of medical education that can be difficult to translate into formal curricula. The role of arts and humanities programs in fostering professional identity formation remains understudied. Analyzing learners’ written reflections, we explore the relationship between an arts-based course and themes of professional identity formation.

**Materials and methods:**

Two cohorts of learners participated in a 5-day online course featuring visual arts-based group activities. Both cohorts responded to a prompt with written reflections at the beginning and end of the course. Using a thematic analysis method, we qualitatively analyzed one set of reflections from each cohort.

**Results:**

Themes included the nature of the good life; fulfilling, purposeful work; entering the physician role; exploration of emotional experience; and personal growth. Reflections written at the end of the course engaged significantly with art – including literature, poetry, lyrics, and film. One student disclosed a mental illness in their reflection.

**Conclusions:**

Our qualitative analysis of reflections written during a visual arts-based course found several themes related to professional identity formation. Such arts-based courses can also enrich learners’ reflections and provide a space for learners to be vulnerable.

**Practice Points:**

(five short bullets conveying the main points)

- Arts-based courses can support learners’ professional identity formation
- Reflection themes related to professional identity formation included entering the physician role, fulfilling clinical work, and personal growth
- At the end of the course, learners’ reflections included significant engagement with art
- Reflective writing in small, arts-based learning communities can provide space for learners to be vulnerable

The Role of Arts-Based Curricula in Professional Identity Formation: Results of A Qualitative Analysis of Learner’s Written Reflections

## Introduction

Professional identity formation (PIF) – the complex process by which medical students learn to think, feel and act like physicians – has become an important focus of medical education [1]. PIF asks students to integrate their personal, social, and professional identities to become physicians who bring knowledge, ethical sensibilities, and emotional intelligence to the care of patients [2]. To best support PIF, medical education must expand from teaching students how to do the work of a physician to the broader task of helping them become a physician [3].

The multi-faceted complexity of PIF [4] – a sociocultural process influenced by the ‘hidden curriculum’ of medical education, socialization into a community, and an individual’s character and experiences – poses challenges for designing curricula intended to shape learner’s professional identities. However, programs that integrate the arts and humanities into medical education have been found to foster professional development and identity formation [5–7] by several means, including enhancing empathy, tolerance for ambiguity, communication, teamwork, and joy [8,9].

Most arts and humanities programs rely on literary arts-based methods (e.g., reading literature, reflective writing, and narrative medicine) [10] to support PIF-relevant learning objectives. A growing number of publications have demonstrated that visual arts-based methods (e.g., creating or observing art, Visual Thinking Strategies [VTS]) can also contribute to PIF-related learning goals [11–14]. A work of art often achieves this by serving as a ‘third thing’ that invites metaphorical thinking and interactive discussion [15,16]. A work of art, combined with a pedagogical method like VTS that encourages vulnerability and builds trust within a learning community, can be a catalyst for exploring learners’ feelings and thoughts, including biases. In our experience, visual arts-based methods have a synergy with literary arts-based methods that allow learners to venture into unfamiliar, even uncomfortable, territory.

Despite the rise in the use of arts-based methods to promote PIF over the past decade, few programs have been rigorously studied [8,10]. To better understand how the combination of visual and literary arts-based pedagogical methods might support PIF, we designed a primarily visual arts-based 1-week course for medical students that incorporated literary methods, including formative and summative writing assignments [17,18]. For these assignments, we asked participants to reflect on ‘big questions’ such as what it means to be human, to be a physician, and to lead a good life. This course and these questions came at a critical time during the Covid-19 pandemic, during which students’ personal and professional identities were challenged by high levels of emotional exhaustion, cynicism, and burnout [19–21].

In this paper, we explore the relationship between visual and literary arts-based methods with PIF by qualitatively analyzing participants’ written responses to reflective prompts in the course. A secondary aim was to compare participants’ reflections written at the start of the course to reflections written at the completion of the course to assess for thematic change.

## Materials & methods

### Course overview

A multidisciplinary team contributed to the development of the course, which has been described in detail previously [18]. The teaching team delivered this full-time 5-day online elective course to second- through fourth-year Johns Hopkins University medical students in April and May 2020. For both cohorts, each day included a 2-hour synchronous online session of primarily visual arts-based group activities. After the first and final days of the course, students responded to a prompt with a 750- to 1500-word written reflection. The prompts are displayed in Table 1. The two cohorts addressed the same prompt (‘What does a good life look like to you, for yourself and your future patients?’) at different points in the course. We refer to the cohorts based on when they encountered the shared prompt (early course or late course).

**Table 1.** Reflection prompts. The shared prompt whose reflections were analyzed in this study is bolded.

	First Cohort (late course cohort)	Second Cohort (early course cohort)
First Day Reflection Prompt	‘What does it mean – to you – to be a physician?’	‘What does a good life look like to you, for yourself and your future patients?’
Final Day Reflection Prompt	‘What does a good life look like to you, for yourself and your future patients?’	‘What does it mean to be human, be a physician, and/or lead good life?’

### Participants and data

We recruited two cohorts of participants from the 18 students enrolled in the two offerings of the course (10 and 8 students, respectively). Following the conclusion and submission of grades for both courses, we contacted students via email to ask whether they would consent to participate in a research study of their narrative reflections. No incentives were provided for participation. The reflections averaged 1136 words. Of 18 total students, all of whom who had completed both written assignments, 10 consented to participate (6 identifying as female, 4 identifying as male). The Johns Hopkins Medicine Institutional Review Board determined that the study qualifies as exempt research.

### Qualitative analysis

Two authors (JA and BM) performed the qualitative thematic analysis of the participants’ narrative reflections as described by 22,using NVIVO 12 qualitative analysis software. JA had graduate school training in qualitative methods and trained the second analyst for the study. Neither of the coding authors were involved with the data collection or the course and had no prior interactions with the students whose reflections they analyzed. Both analysts had experience with medical education and interests in reflective writing.

Given that our qualitative analysis spanned two cohorts of essays and our research question was whether there were thematic differences between the two, the qualitative analysts performed two analyses in parallel. They created separate codebooks and themes for each group of essays. During the analysis, the coders were blinded to which group of essays were written before or after the course.

In Phase 1 of the study, the two qualitative analysts familiarized themselves with the data by reading the essays several times over. In Phase 2, they coded each of the essays in stepwise fashion using codes that inductively emerged from the text (codes were not set a priori). They separately coded the first two essays of each cohort using constant comparison, then met to reconcile and define codes before proceeding to code the remaining essays. Both authors reconciled codes for the remaining essays. In Phase 3, they finalized the codebook and organized codes into categories based on conceptual similarity between codes. They then independently reviewed the essays a second time to ensure standardized application of codes. Both authors agreed on any revisions. The authors implemented this process of independent coding, reconciliation, and repeated review of the original text to limit an individual researcher’s perspective on the analysis and to maintain grounding in the reflections. In Phase 4, the qualitative analysts worked collaboratively to draft a set of themes derived inductively from the codebook categories and fleshed into major sub-themes as appropriate. They then compared the draft themes to the primary texts associated with each theme’s constituent codes and refined the themes as necessary. They then reviewed the entire data set with the themes in mind, and themes were adjusted as appropriate to again reflect the original text. In Phase 5, the authors defined and named the resulting themes. In Phase 6, they reported the themes as a part of writing this manuscript. Throughout this process, the coding authors retained independence from the other authors. As a final check on the analysis, the findings were discussed with the other authors who approved the consistency of the findings with their readings of the reflections and with relevance to the original research aims.

We wrote the manuscript to align with journal article reporting standards for qualitative primary, qualitative meta-analytic, and mixed methods research in psychology (JARS-QUAL), as applicable [23].

## Results

The analysis of each cohort’s reflections resulted in three themes each, for a total of six themes. The early-course reflection themes (see Table 2) comprise: the nature of the good life, entering the physician role, and exploration of emotional experience. The late-course reflection themes were: the nature of the good life; fulfilling, purposeful work; and personal growth. Each theme is presented below, and when there was significant overlap between the early- and late-cohorts (e.g., both wrote significantly on the nature of the good life), they are presented in the same section. Four of the six themes were different between the early- and late-courses.

**Table 2.** Themes from the two cohorts.

Themes from the early-course reflections	Themes from the late-course reflections
The nature of the good life	The nature of the good life
Entering the physician role	Fulfilling, purposeful work
Exploration of emotional experience	Personal growth

### The nature of the good life (both cohorts)

At least two participants in the early-course cohort described uncertainty about the definition of the good life and its consistency between persons.

*“I know what life is, but I wonder, what is “good”?” (early-course cohort, essay 1)*

*“It is hard to say that one thing or word is wholly good. I believe that a good life can only be defined contextually” (early-course cohort, essay 3)*

*“What is a good life for my patient is what my patient wishes for that life to be. And I say that … to emphasize each patient’s individuality and to de-emphasize my ability to surmise what their view of “good” and “life’ and a “good life” might be.” (early-course cohort, essay 3)*

At least one person from the early-course cohort suggested that there might be some consistency in perspectives on the good life between a physician and their patients:

*“Happiness, peace and balance, I think, are the most important things for me to live a good life, and therefore, for my patients to live a good life as well.” (early-course cohort, essay 2)*

Several participants in the late-course cohort also addressed the theme of consistency between persons, with many students noting that there were plural conceptions of the good life:

*“A good life can manifest in an infinite variety of ways.” (late-course cohort, essay 3)*

*“Since a good life is subjective, some may say that it is not a fair exercise to categorize people’s lives into good and bad … My patients have their own autonomy to determine what a good life is to them.” (late-course cohort, essay 4)*

*“My role as a physician will involve creating relationships with patients to help them achieve their own understanding of what a good life looks like to them.” (late-course cohort, essay 6)*

Altogether, both cohorts largely endorsed a plural, subjective nature of tolerance for ambiguity.

Participants from the late-course cohort acknowledged different practices, traditions, and fields of thought to ground their understanding of the good life. These included poetry (essay 6), meaningful quotations (essay 2), philosophical thought (essays 3, 4), religious traditions (essays 3, 4), mindfulness practices (essay 5), nature (essay 6), and artwork (essay 6).

Each cohort described the importance of material, external factors (e.g., financial security, food, shelter, basic needs) as well as internal dispositions (e.g., traits such as gratefulness, curiosity, and mindfulness and emotions such as happiness, peace, and comfort) to achieving a good life. Several students from the early-course felt it was important to find components of the good life that were timeless – cherishing meaningful moments even before they’ve reached their career goals. Participants from the late-course reflections described the importance of religion, nature, community and meaningful relationships in transcending an individual idea of the good life.

### Fulfilling, purposeful work (late-course cohort)

The late-course cohort explored their desire to participate in fulfilling, purposeful work in their future. Thinking about a future in psychiatry, the author of essay 2 explains why they see this as fulfilling work:

*“You get to give people back one of the most central elements of themselves, their mind … I find it difficult to think of a more fulfilling pursuit … My dream is to sustainably assist my patients as they pursue their own good life.” (late-course cohort, essay 2)*

Many students identified different purposes of their future work as a physician including helping others, preventing suffering, promoting health, caring for others, serving others, empowering patients, and contributing to their broader community.

### Entering the physician role (early-course cohort)

Similarly, the early-course cohort reflected on entering the physician role. One participant devoted a significant portion of their reflection on the fulfilling aspects of their future work, but voiced the fear:

*“will I continue to be fulfilled as my career progresses?” (early-course cohort, essay 4)*

Many participants from the early-course cohort describe specific career goals including to be a pleasant doctor (essay 1), to enable patients (essay 1), to build strong relationships with their patients (essay 2), to help others (essay 4), a specific specialty choice (essay 4), and to help patients find their good life (essay 4).

### Exploration of emotional experience (early-course cohort)

Many participants in the early-course cohort described emotional experiences. A particular focus was the idea of happiness and its connection to relationships, business, memories, financial security, and the good life. One essay (essay 2) extensively described an emotionally difficult time in early medical school when they felt lost, unhappy, afraid, inadequate, judged, criticized, pressured, and excluded to the point that they were diagnosed with clinical depression. They described a process of growth in which they were able to get professional help and learn to start to accept themselves.

### Personal growth (late-course cohort)

The late-course cohort thoroughly described key components of personal growth including recognition of one’s weaknesses, the desire for growth, and methods of change. Essay 5 captures aspects of each in relation to their search for the good life:

*“For a long time, I equated a good life with the perfect life. I had a plan with specific goals regarding how I wanted my life to look … There was very little time spent in the present and appreciating what was actually going on whether positive or negative. I realized that the perfect life wasn’t all that good … Consequentially, I have made a lot of substantial changes to how I approach many aspects of my life, especially with regards to taking steps to stop and just be present in whatever moment I am currently experiencing … ” (late-course cohort, essay 5)*

Students mentioned life experience, nature, mindfulness, patient encounters, personal relationships, and inspirational quotations as resources to catalyze personal growth.

## Discussion

Our descriptive, qualitative analysis explored how a visual arts-based curriculum that incorporates literary arts methods might support PIF.

Regarding our primary aim – scrutinizing learners’ engagement with themes related to PIF – both cohorts wrote extensively about personal growth, entering the physician role, and purposeful work. These themes addressed core components of PIF: introspection, emotional awareness, self-assessment and contemplation of role transitions [24–26]. Educators who want to foster PIF with reflective exercises might orient reflection prompts and discussions around these topics. Our work bolsters the growing literature linking reflective practices to PIF [26,27]. In addition, several themes in the students’ essays related to developing personal insight, embodying one of the four approaches to the arts and humanities in medical education as described by the Prism Model [28,29]: skill mastery, perspective taking, personal insight, and social advocacy.

Regarding our secondary aim of identifying differences between the two cohorts, the visual arts basis of the curriculum appeared to enrich students’ reflections. The cohort whose reflections were written at the end of the course (the ‘late-course’ cohort) demonstrated significant engagement with various ‘third objects’ (e.g., photographs, paintings, imagery from nature) as reported in both the nature of the good life and personal growth themes. These objects allowed learners to encounter and develop new insights about the good life and their own personal growth. Learner’s engagement with these objects and their subsequent self-discoveries demonstrate both the personal insight and perspective taking approaches described by the Prism Model [28,29].

One unexpected result was a student’s written disclosure of mental illness, embedded in a reflection, to the course director. The student acknowledged that she was not in crisis currently and was receiving the help she needed but did share private information about her struggles in medical school. Medical students and physicians have historically under-disclosed mental illness, which discourages care-seeking [30,31]. To address this, educators have called for promotion of vulnerability, personal disclosure from faculty, and cultivation of a professional culture that values wellbeing [32]. One student’s disclosure of mental illness in the written exercise suggests that the course was able to foster a safe learning environment in which students could express vulnerability. Reflective writing might serve as a venue in the formal curriculum for interpersonal vulnerability and community building, particularly in group settings [33], which can promote care-seeking and support for mental illness [30,34]. Educators should be prepared to respond to such disclosures by students, especially when the student is still struggling with an episode of mental illness.

We acknowledge limitations to our work. First, several factors limited the generalizability of our findings. This was a small cohort of students, at a single site, and participation in the course and inclusion of the reflection in our analysis were optional for students. Students who did not participate in the course or did not allow their reflection to be analyzed might have different perspectives than those who did participate. Second, we did not directly ask students about how this course affected their professional growth, though they indirectly addressed those themes in their reflections. Students might have different thoughts if asked directly. Finally, associations or trends in particular groups might reflect the small, self-selected samples rather than meaningful differences between the two produced by the course.

## Conclusion

Our qualitative analysis of student writings suggests that participation in a visual arts-based course that incorporates written reflective practice can support students’ PIF, perspective taking, and development of personal insight. Such arts-based courses can also allow for expression of vulnerability in supportive learning communities.

## Data Availability

The reflections are saved in the course records and are not available publicly online. The anonymized reflections were made available to the qualitative analysts for the purpose of this study.
